# The subplacenta of the red-rumped agouti *(Dasyprocta leporina L)*

**DOI:** 10.1186/1477-7827-4-31

**Published:** 2006-06-01

**Authors:** Rosangela Felipe Rodrigues, Anthony M Carter, Carlos Eduardo Ambrosio, Tatiana Carlesso dos Santos, Maria Angelica Miglino

**Affiliations:** 1 Department of Surgery, School of Veterinary Medicine, University of Sao Paulo, Sao Paulo, Brazil; 2 Department of Physiology and Pharmacology, University of Southern Denmark, Odense, Denmark

## Abstract

**Background:**

Hystricognath rodents have a lobed placenta, comprising labyrinthine exchange areas and interlobular trophoblast. These correspond to the labyrinthine and spongy zones of other rodent placentae. Beneath them, however, is a structure unique to hystricognath rodents called the subplacenta. We here describe the subplacenta of the red-rumped agouti and examine the possible functional correlates of this structure.

**Methods:**

Placentae were collected from early in midgestation to near term of pregnancy and examined by standard histological techniques, immunohistochemistry and transmission electron microscopy. In addition, to study the microvasculature of the subplacenta, vessel casts were inspected by scanning electron microscopy

**Results:**

In the subplacenta, lamellae of connective tissue support a layer of mononuclear cytotrophoblast cells. Beneath this is found syncytiotrophoblast. Clusters of multinuclear giant cells occur in the transition zone between the subplacenta and decidua. There are prominent intercellular spaces between the cytotrophoblast cells. The basal membrane of these cells is often close to fetal blood vessels. The syncytiotrophoblast surrounds an extensive system of lacunae. Microvilli project into these lacunae from the plasma membrane of the syncytiotrophoblast. The syncytial cytoplasm contains electron-dense granules. This is probably the amylase-resistant PAS-positive material identified by histochemistry. The subplacenta is supplied entirely from the fetal circulation. Within it the vessels pursue a tortuous course with sinusoidal dilatations and constrictions.

**Conclusion:**

The functions that have been attributed to the subplacenta include hormone production. Our findings are consistent with this interpretation, but suggest that hormone secretion is directed towards the fetal circulation rather than the maternal tissues.

## Introduction

The hystricognath rodents (Suborder Hystricomorpha, Infraorder Hystricognathi [1]) appeared in the Eocene and underwent an extensive radiation in the Miocene. At this time they were able to capitalize upon the emergence of grasslands for which they were well adapted in a number of ways [2]. They differ from other rodents in giving birth to precocial young. The newborn is well developed with open eyes and a full coat of hair [3]. This reproductive strategy requires a lower rate of energy consumption and is well suited to an herbivorous diet [2]. The hystricognath placenta has a number of distinctive features [4,5]. The exchange area or labyrinth is lobulated, an adaptation that allows an increase in the total exchange area and helps to support the larger fetus at the end of gestation [6]. The lobules are separated by interlobular trophoblast that is the counterpart of the spongy layer found in the placenta of other rodents. Beneath this is a structure known as the subplacenta that is unique to the hystricognath rodents.

In this paper we review the structure of the subplacenta and examine possible functional correlates. The analysis is based on a description of the placenta of the red-rumped agouti (*Dasyprocta leporina*). This is a medium sized rodent, larger than a guinea pig and with longer legs. It is found throughout the forest, where it lives mainly on fallen fruits and nuts. Agoutis usually sit erect to eat, holding the food in their hands. They bury excess nuts and fruits for use when food is scarce. Because they bury them singly (scatter hoarding) rather than many in a cache, they are important seed dispersers for a number of tree species [7,8].

The functions that have been ascribed to the subplacenta include hormone production [9,10]. Our findings are consistent with this interpretation, but suggest that hormone secretion is directed towards the fetal circulation. Trophoblast giant cells occur close to the margin of the subplacenta and we included these in our analysis. We note that, unlike the trophoblast of the subplacenta, the giant cells often occur in close proximity to maternal blood vessels.

## Materials and methods

The study was based on 9 placentae from 6 red-rumped agoutis collected from early in midgestation to near term of pregnancy (Table 1). The research was authorized by the Brazilian Institute of Environment and Renewable Natural Resources (IBAMA). The experimental protocol was approved by the bioethics committee of the School of Veterinary Medicine, University of São Paulo. The samples were collected at an agouti breeding facility at São José do Rio Preto, São Paulo. Pregnant females were submitted to hemihysterectomy. Details of anaesthesia and surgical procedures are given elsewhere [11].

**Table 1:** Measurements of fetus, umbilical cord and placenta in the specimens of red-rumped agouti used for the present study.

Animal No.	Stage of gestation	Uterine horn	Fetal length (cm)	Fetal weight (g)	Umbilical cord length (cm)	Placental weight (g)
1	Early to mid-gestation	Left	---	---	---	15
		Left	2	15	2	13
2	Early to mid-gestation	Right	2	15.5	2	12
3	Midgestation	Right	10	42	9.5	37
		Left	10	42	9	36
4	Midgestation	Left	10	40	9	34
5	Midgestation	Right	10.5	41	9.5	36
6	Term gestation	Right	16	100	13	78
		Left	12.5	76	9	50

Placental fragments were fixed in 10% formalin in 0.1 M phosphate buffer and processed by standard histological procedures for embedding in paraplast, and then sectioned at 5 μm (automatic microtome, Model RM2155, Leica, Germany). Sections were stained with haematoxylin and eosin, Masson's trichrome stain or by the periodic acid Schiff (PAS) reaction with and without pretreatment with 1% amylase (Sigma, St Louis, Missouri, U.S.A.) at 37°C for 30 min. The latter sections were counterstained with haematoxylin.

Immunohistochemistry was performed for cytokeratin (to identify epithelial cells and trophoblasts) and vimentin (to identify mesenchymal cells and stromal decidua). For this purpose, 5 μm sections were transferred to poly-L-lysine coated slides. Endogenous peroxidase was blocked with 0.1% hydrogen peroxide. To improve antigen retrieval, the sections were then treated in a microwave oven in 0.1 M Tris-HCl buffer, pH 7.4. To prevent non-specific binding, slides were blocked with 2% milk powder in phosphate-buffered saline for 20 min. They were incubated overnight with primary antibody at 4°C; either a rabbit polyclonal antibody against cytokeratin (1:500; PU071-UP, Biogenex, San Ramon, California, U.S.A.) or a goat polyclonal antibody against vimentin (1:500; SC -1226, Santa Cruz Biotechnology, Santa Cruz, California, U.S.A.). Immunostaining was then performed using a secondary antibody from a kit (LSAB-HRP Peroxidase, Dako, Carpinteria, California, U.S.A.) with diaminobenzidine in Tris-HCl buffer, pH 8.2, as the chromogen. The sections were counterstained with Harris's haematoxylin.

The description of the ultrastructure of agouti subplacenta is based on material from the middle of gestation. For transmission electron microscopy, small samples were fixed in 2.5% glutaraldehyde in 0.1 M phosphate buffer, pH 7.4, washed in buffer and post-fixed in phosphate-buffered osmium tetroxide, pH 7.4 (Polysciences, Warrington, PA, USA). They were then dehydrated, washed with propylene oxide and embedded in Spurr's resin (Spurr's Kit, Electron Microscopy Sciences, Fort Washington, PA, U.S.A.). Sections were made at 60 nm and stained with 2% uranyl acetate (5 minutes) and 0.5% lead citrate (10 minutes). The ultrastructural observations were made with a transmission electron microscope (JEOL 1010, Peabody, MA, U.S.A).

To study the microvasculature of the subplacenta, an umbilical artery was injected with Mercox™ CL-2R (Okenshoji Co., Ltd, Tokyo, Japan) as previously described [11]. Tissues were digested by immersion of the preparation in several changes of 20% NaOH solution at 50–60°C. The casts were rinsed thoroughly in distilled water and dried in an oven at 37°C. They were then refrigerated in 20% gelatin. For scanning electron microscopy, pieces of the casts were rinsed in distilled water to remove the gelatin, dried, and mounted on stubs with conductive carbon cement (Neubauer, Münster, Germany). They were then coated with gold using a sputter coater (Model K550, Emitech Products Inc., Houston TX, USA) and examined in a scanning electron microscope (Model 435 VP, Leo Electron Microscopy Ltd, Cambridge, UK).

As a further aid to understanding vessel distribution, some placentae were injected with coloured latex (Neoprene 650, DuPont, Brazil; Latex Stain, Suvinil, Glassurit do Brazil S/A, São Bernardo do Campo, S.P., Brazil). Different colours were injected in a uterine vein, a uterine artery, and the umbilical vein. The placentae were fixed in 10% formalin in 0.1 M phosphate buffer.

## Results

### General structure

**Figure 1:**
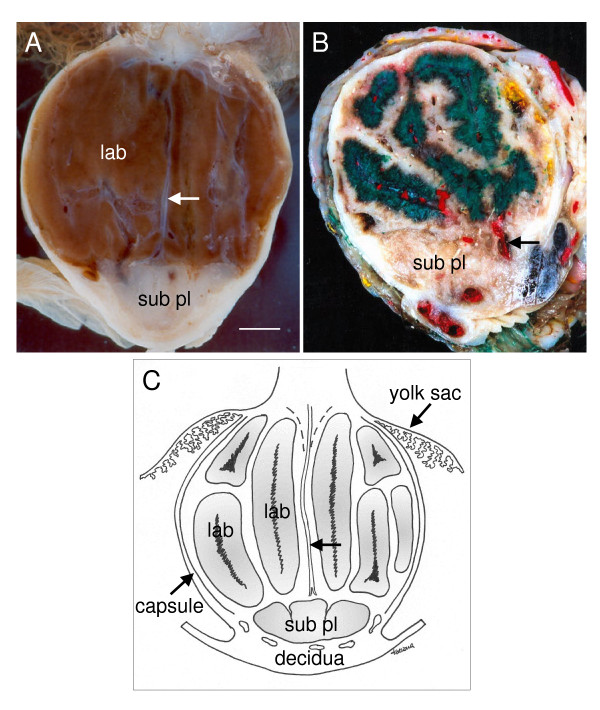
Gross structure of the agouti placenta. (A) Transverse section of the placenta in midpregnancy showing the labyrinth (lab) of the main placenta and the subplacenta (sub pl). Fetal vessels run from the umbilical cord to the subplacenta through the central excavation (arrow). Scale bar: 1 cm. (B) Placenta injected with Neoprene latex to show the vascularization of the subplacenta. Fetal arteries are in white, while maternal arteries in the decidua (arrow) are in red; maternal venous channels were filled with green latex. (C) Schematic to show location of the subplacenta in relation to the yolk sac placenta, main placenta and decidua. The labyrinth occurs in lobes separated by interlobar trophoblast. In addition to the decidua beneath the subplacenta, maternal tissue forms a capsule that houses the placenta. The trajectory of the fetal vessels is also indicated (arrow).

The placenta is found on the mesometrial side of the uterus. In transverse section the main placenta and the subplacenta can be distinguished by their colour and form (Figure 1A). The placenta is spherical and reddish and on closer inspection is seen to be divided into lobes separated by interlobar trophoblast (Figure 1C). The subplacenta is whitish and formed like a chalice (Figure 1A). Beneath it is maternal decidua. Giant cells occur in the transition zone between the subplacenta and decidua. Maternal tissue forms a capsule that encloses the placenta (Figure 1C).

In midgestation the subplacenta is supplied entirely by fetal blood vessels. Thus a single large artery runs through the central excavation from the umbilical cord to the subplacenta (Figure 1A, C). When Neoprene latex is injected into the umbilical artery, it fills the vessels of the subplacenta (white colour in Figure 1B). In contrast, latex injected through the uterine arteries does not reach the subplacenta, although it fills large maternal vessels peripheral to it (red colour in Figure 1B).

### Histology and immunohistochemistry

**Figure 2:**
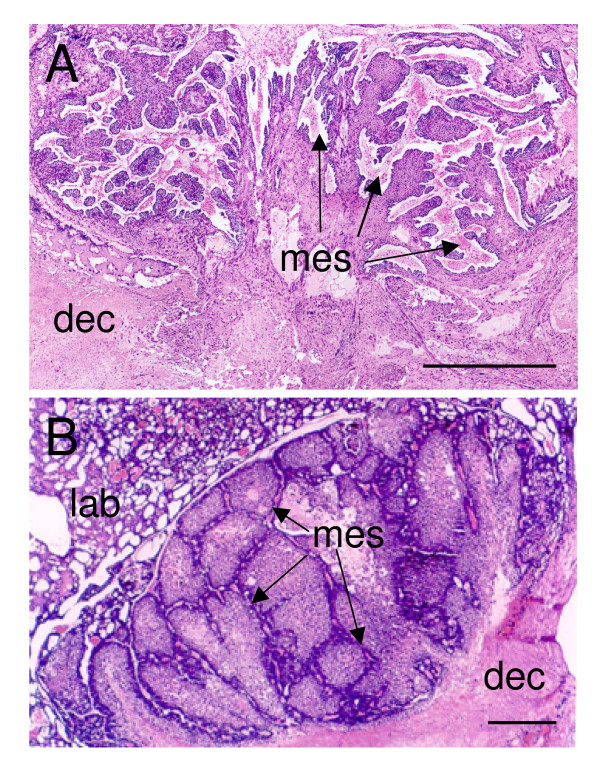
Subplacenta of the agouti. (A) Early in gestation. Note the lamellae of fetal mesenchyme (mes) and the relationship of the subplacenta to the decidua (dec). (B) Later in gestation. The lamellae of fetal mesenchyme are much thinner; lab, labyrinth. Haematoxylin and eosin. Scale bars: 0.5 mm.

The subplacenta is separated from the main placenta by a layer of connective tissue (fetal mesenchyme), and lamellae of connective tissue support the trophoblast. This is clear early in gestation when there is a relatively open structure with plenty of connective tissue (Figure 2A). Later the structure becomes more compact and lobular with thinning of the connective tissue (Figure 2B). The subplacenta does persist until the end of gestation, but extensive degenerative changes occur towards term (not shown).

**Figure 3:**
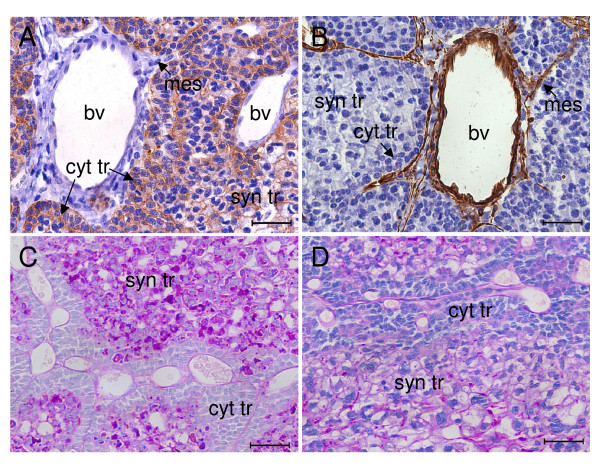
Immunohistochemistry and PAS reaction of agouti subplacenta in midgestation. (A) Cytotrophoblast (cyt tr) and syncytiotrophoblast (syn tr) immunostain for cytokeratin. Fetal mesenchyme (mes) and wall of blood vessel (bv) are cytokeratin negative. (B) Fetal mesenchyme and wall of blood vessel immunostain for vimentin. Trophoblast is vimentin negative. (C) PAS reaction in the absence of amylase is strong in syncytiotrophoblast and negative in cytotrophoblasts (although their basal lamina gives a positive reaction). (D) Following treatment with amylase there is only a moderate decrease in the positive reaction of syncytiotrophoblast. Scale bars: 40 μm.

**Figure 4:**
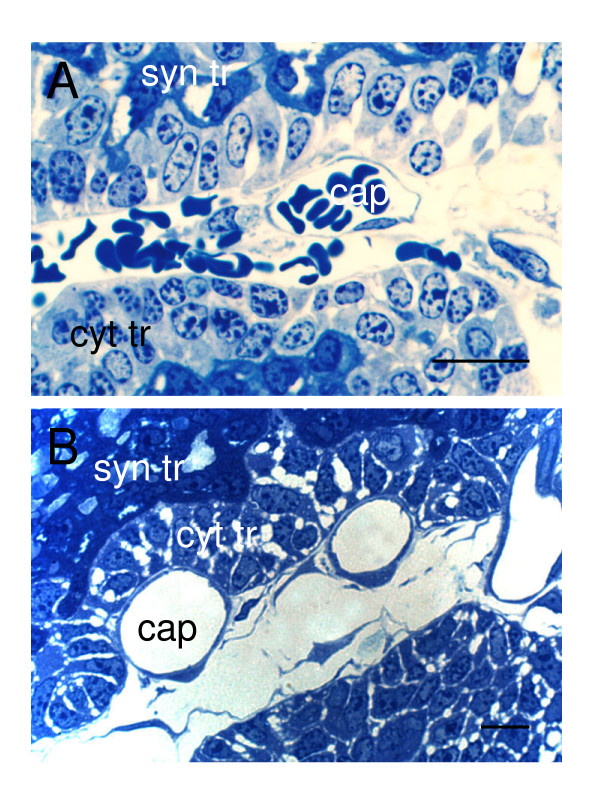
Organization of the agouti subplacenta. The cytotrophoblast (cyt tr) abuts the mesenchyme and fetal capillaries (cap). Behind it is found syncytiotrophoblast (syn tr). (A) Early in gestation. (B) Later in gestation. Semithin sections, toluidine blue. Scale bars: 20 μm.

As might be expected the trophoblast can be immunostained for cytokeratin (Figure 3A). The connective tissue carries the fetal blood vessels. The latter are cytokeratin negative (Figure 3A), but they can be immunostained for vimentin (Figure 3B). The connective tissue lamellae are bordered by a layer of mononuclear cytotrophoblast with clearly marked cell boundaries (Figure 4). Beneath this is syncytiotrophoblast with a completely different morphology: multinucleate and without cell boundaries. The cytotrophoblasts have basophilic cytoplasm, round nuclei and rest on a basal membrane. The cytoplasm of the syncytiotrophoblast is eosinophilic, with basophilic granules, and the nuclei are irregular.

The syncytiotrophoblast is PAS-positive and PAS-positive granules are present in the cytoplasm (Figure 3C). The PAS reaction persists after amylase treatment (Figure 3D). The most intense staining is likely related to the lacunae that occur in the syncytiotrophoblast, as described below.

With the progress of gestation some initial characteristics of the subplacenta change. Thus there is a reduction in the number of the layers of cytotrophoblast, which initially is multi-layered (Figure 4A) and later forms a single layer in most places (Figure 4B). In the syncytiotrophoblast, the advance of gestation is marked by the appearance of extensive lacunae. Towards term, as the subplacenta starts to degenerate, one sees vacuolization of the syncytiotrophoblast.

**Figure 5:**
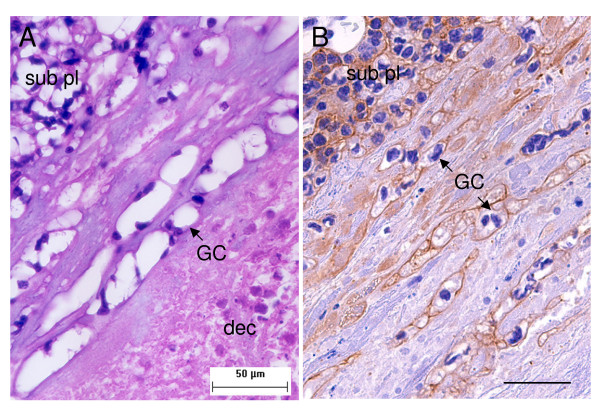
Trophoblast giant cells of agouti placenta in midgestation. (A) Clusters of multinucleated giant cells (GC) are found in the transition zone between the subplacenta (sub pl) and decidua (dec). (B) The giant cells can be immunostained for cytokeratin. Scale bars: 50 μm (A); 40 μm (B).

Clusters of multinucleated giant cells are found near the margin of the subplacenta in the transition zone between it and the decidua (Figure 5A). They often occur in proximity to maternal blood vessels. The giant cells have clear and irregular cytoplasm with round nuclei and are surrounded by eosinophilic and PAS-positive extracellular material. They can be immunostained for cytokeratin (Figure 5B).

### Ultrastructure

**Figure 6:**
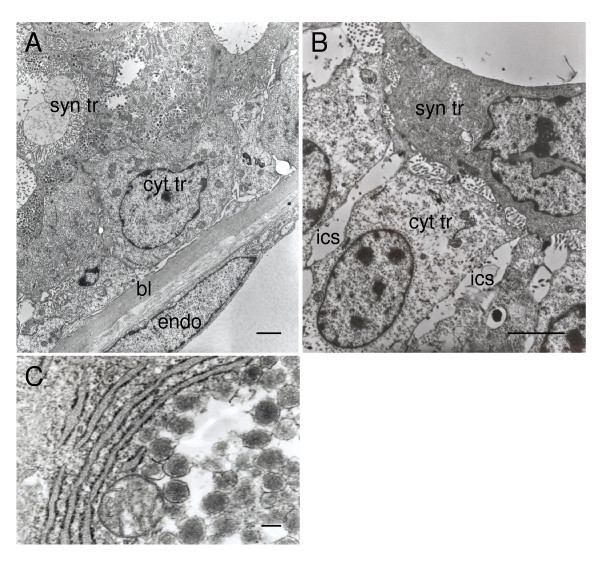
Ultrastructure of cytotrophoblast cells of the agouti subplacenta in midgestation. (A) Cytotrophoblasts (cyt tr) rest on a basal lamina (bl) that is closely apposed to the endothelium of a fetal capillary. On the other side they are in contact with the syncytiotrophoblast (syn tr). (B) Large intercellular spaces (ics) occur between the cytotrophoblasts. (C) Detail showing abundant rough endoplasmic reticulum and numerous electron dense inclusions. Scale bars: 2 μm (A); 3 μm (B); (C) 200 nm.

The nuclei of the cytotrophoblast cells are large in relation to the amount of cytoplasm and have euchromatin with an evident nucleolus (Figure 6A). The basal membrane of the cytotrophoblast layer is in contact with the connective tissue lamellae and often close to the fetal vessels (Figure 6A). The lateral membrane shows microvilli in some places. There are often large intercellular spaces (Figure 6B). The cytoplasm contains rounded mitochondria, rough endoplasmic reticulum and small electron dense inclusions (Figure 6C). Desmosomes are seen between cytotrophoblast cells as well as between cytotrophoblast and the underlying syncytiotrophoblast.

**Figure 7:**
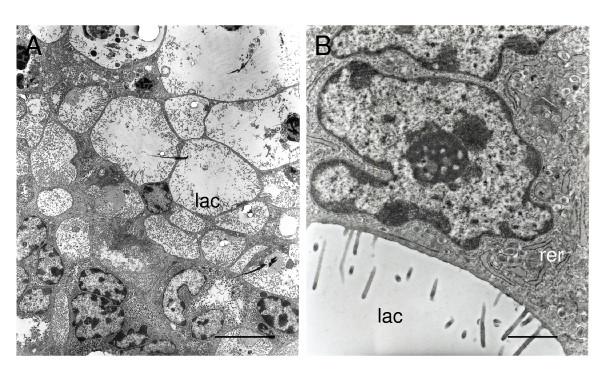
Ultrastructure of syncytiotrophoblast of agouti subplacenta in midgestation. (A) Syncytiotrophoblast sends microvillous projections into an extensive system of lacunae (lac). (B) Detail showing irregularly shaped nucleus, rough endoplasmic reticulum (rer) and vesicles near the plasma membrane. Scale bars: 5 μm (A); 1 μm (B).

The syncytiotrophoblast encloses many lacunae into which microvilli project from the plasma membrane (Figure 7A). As gestation advances, these lacunae come to form an extensive system. The syncytiotrophoblast has many irregular nuclei with euchromatin and evident nucleoli (Figure 7B). The cytoplasm contains few mitochondria, some rough endoplasmic reticulum, and an occasional Golgi apparatus. Throughout the cytoplasm it is possible to identify electron dense granules.

**Figure 8:**
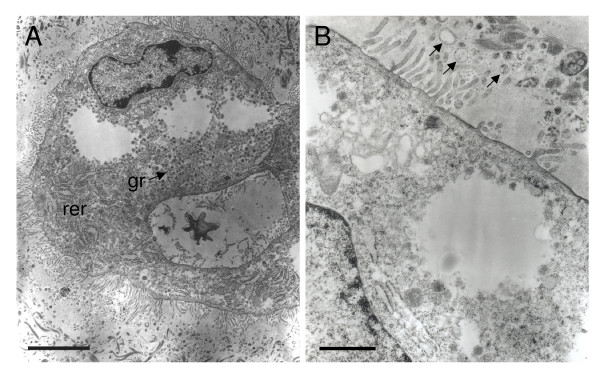
Ultrastructure of a placental giant cell in the agouti in midgestation. (A) The nucleus is irregular and the cytoplasm appears vacuolated. There are few mitochondria, some rough endoplasmic reticulum (rer) and many granules (gr) of moderate electron density. Microvilli extend from the cell surface. (B) At higher power vesicles (arrows) can be seen within the extracellular material. They seem to have been extruded from the cell. Scale bars: 4 μm (A); 1 μm (B).

The giant cells have irregular nuclei and their cytoplasm appears vacuolated (Figure 8A). They contain few mitochondria and the rough endoplasmic reticulum is rather sparse, but there are many granules of moderate electron density. Microvilli extend from the cell surface into the surrounding matrix. Vesicles seen within this extracellular material seem to have been extruded from the cell (Figure 8B).

### Microvasculature

**Figure 9:**
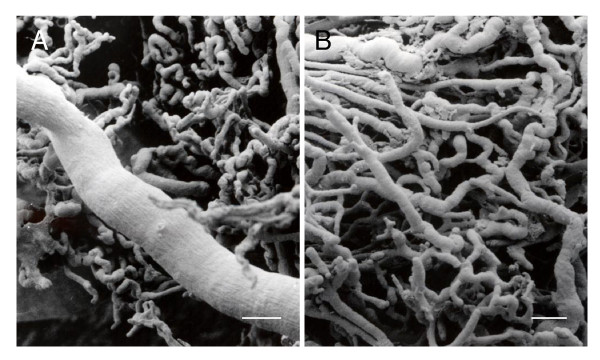
Microvasculature of agouti subplacenta in midgestation. (A) The large vessel is a branch of the umbilical artery. (B) A skein of vessel branches. Note the sinusoidal dilatations and constrictions of the capillaries. Scale bars: 100 μm.

In mid to late gestation, the subplacenta is supplied exclusively by fetal vessels (Figure 1B). A large branch of the umbilical artery follows the central band of fetal mesenchyme to the base of the main placenta and then branches to supply the subplacenta (Figure 9A). The subplacental vessels pursue a tortuous course with sinusoidal dilatations and constrictions (Figure 9B).

## Discussion

The subplacenta is a unique structure that distinguishes hystricognath rodents from all other mammals [4,5]. The basic arrangement of cytotrophoblast and syncytiotrophoblast, supported by lamellae of connective tissue, is well conserved. The ultrastructure of these elements is also similar across species, including the guinea pig [9,10,12], chinchilla [13], cane rat [14], paca [15], rock cavy [16] and agouti (this study). The function of the subplacenta is poorly understood.

In the guinea pig, fetal trophoblast invades the walls of the uterine arteries and this is associated with extensive remodelling of the vessel wall [17,18]. Recently, it was proposed that the source of the invasive trophoblast was the cytotrophoblast layer of the subplacenta [19]. There is experimental evidence from the guinea pig and degu to support this hypothesis [20,21]. It does not, however, address the function of the syncytiotrophoblast. An earlier proposal that the subplacenta might play a role in maternal to fetal transfer of high molecular weight molecules [22] receives no support from studies of its ultrastructure [12,13].

The placenta is a source of steroid hormones. In the guinea pig ovarian progesterone is not required after day 20 of gestation, implying that placental progesterone is adequate for pregnancy maintenance after this time [23]. It has been shown for a variety of hystricognath rodents that the hormone circulates bound to progesterone-binding protein [24]. The principal site of placental progesterone synthesis is the interlobar syncytiotrophoblast [25]. This is supported by the presence there of smooth endoplasmic reticulum and mitochondria containing tubular cristae [26]. In contrast Wolfer and Kaufmann [12] argued against a role for the subplacenta in steroid synthesis. Attention has therefore been focussed on the subplacenta as a source of glycoprotein.

Davies et al. [9,10] remarked upon the strongly positive PAS reaction of the guinea pig subplacenta. This is in part due to the presence of glycogen, but there is a diastase-resistant component, as demonstrated here for the agouti. It was suggested that this was glycoprotein and that it was secreted into the lacunae [9,10]. Heap and Illingworth [27] proposed that it might be the progesterone-binding protein, as this is a carbohydrate-rich transport protein. The association of the subplacenta with a transport protein likewise unique to hystricognath rodents constituted an attractive hypothesis. However, the cellular location of progesterone-binding protein was later shown to be the interlobar and marginal trophoblast of the main placenta [28].

Davies et al. [9,10] had implied that the glycoprotein might be chorionic gonadotropin (CG), but the evidence for this is equivocal. Indeed, recent work suggests that CG is restricted to primates and equids [29,30]. There is some evidence for production of luteinising hormone, possibly CG, in guinea pig placenta [31], but no detectable signal for the corresponding messenger RNA was seen in Northern blot assays of placental RNA [32]. In primates, CG is secreted directly into the intervillous space from the maternal-facing syncytiotrophoblast, whilst in equids CG is secreted to maternal blood from the endometrial cup cells. Thus, if there is a guinea pig CG, it is more likely to be produced by the syncytiotrophoblast of the main placenta than the subplacenta as it can then be secreted to the maternal blood channels. As we have shown here, the blood supply to the subplacenta is derived from the fetal circulation. A longitudinal study in the guinea pig showed that maternal vessels were present only early in development and had disappeared by day 20 of gestation [12].

The subplacental syncytiotrophoblast contains electron dense inclusions that likely are secretion granules as argued by Wolfer and Kaufmann [12]. Their product is released into the elaborate network of lacunae. These do not have access to the maternal circulation, but King and Tibbitts [13] suggested that products secreted to the extracellular spaces might be able to reach the fetal capillaries. As we show here, the fetal vessels within the subplacenta pursue a tortuous course with dilatations and constrictions as in an endocrine gland.

Secretion of most hormones produced by the human placenta is unidirectional: they are released to the maternal circulation and affect maternal physiology. However, some placental products do reach the fetal circulation. In sheep they include prostaglandin E_2 _[33], adenosine [34] and steroids [35]. As an example, fetal breathing movements occur throughout most of gestation, but are influenced by prostaglandin E_2 _from the placenta, which tends to suppress them. The removal of the placenta and the placental prostaglandin E_2 _is critical for the initiation of continuous breathing at birth [33]. Thus, if the subplacenta is an endocrine organ, the function of its hormones may be to influence fetal physiology.

The origin of the trophoblastic giant cells was explored by Mossman [36]. They tend to occur in clusters surrounded by amorphous extracellular material, as described here for the agouti and elsewhere for the chinchilla [13]. Electron dense granules were seen both within the cells and in the extracellular matrix. This is consistent with a secretory function as suggested in a study of chinchilla giant cells by Tibbitts and Birge [37]. The giant cell clusters sometimes occur close to maternal vessels. Thus, in contrast to those of the subplacenta, their secretory products may have access to the maternal circulation. On the other hand, many giant cells are vacuolated; this has been considered to be a degenerative change [37].

In conclusion, the function of the subplacenta remains elusive. The syncytiotrophoblast secretes PAS-positive material, probably glycoprotein, to an extensive system of lacunae. These lacunae seem to be connected to the intercellular spaces of the cytotrophoblast layer, which lie directly beneath fetal capillaries. Indeed, the subplacenta is plentifully supplied with blood vessels from the umbilical circulation, but lacks direct access to the maternal circulation. Perhaps the function of the subplacenta is to secrete growth factors, hormones or cytokines to the fetal circulation. As in the sheep, they might function to shut down physiological functions that are not needed in fetal life. Placental signals disappear once the cord is severed, allowing immediate resumption of suppressed functions in the neonate. Like sheep, hystricognath rodents give birth to precocial young and we suggest that placental control of fetal function is an important feature of this reproductive strategy.

## References

[B1] Wilson DE, Reeder DM (2005). Mammal Species of the World: A Taxonomic and Geographic Reference.

[B2] Mess A, Mohr B, Martin T (2001). Evolutionary transformations of hystricognath Rodentia and the climatic change in the Eocene to Late Oligocene time interval. Mitt Mus Natkd Berlin Zool Reihe.

[B3] Enders RK (1931). Parturition in the agouti, with notes on several pregnant uteri. J Mammol.

[B4] Luckett WP, Mossman HW (1981). Development and phylogenetic significance of the fetal membranes and placenta of the African hystricognathous rodents *Bathyergus *and *Hystrix*. Am J Anat.

[B5] Mess A (2003). Evolutionary transformations of chorioallantoic placental characters in rodentia with special reference to hystricognath species. J Exp Zoolog Part A Comp Exp Biol.

[B6] Mossman HW, DeHaan RL, Ursprung H (1965). The principal exchange vessels of the chorioallantoic placenta of mammals. Organogenesis.

[B7] Forget PM (1990). Seed-dispersal of Vouacapoua americana (Caesalpinaceae) by caviomorph rodents in French Guiana. J Tropical Ecol.

[B8] Henry O (1999). Frugivory and the importance of seeds in the diet of the orange-rumped agouti (*Dasyprocta leporina*) in French Guiana. J Tropical Ecol.

[B9] Davies J, Dempsey EW, Amoroso EC (1961). The subplacenta of the guinea pig: an electron microscopic study. J Anat Lond.

[B10] Davies J, Dempsey EW, Amoroso EC (1961). The subplacenta of the guinea-pig: development, histology and histochemistry. J Anat Lond.

[B11] Miglino MA, Carter AM, Ambrosio CE, Bonatelli M, De Oliveira MF, Dos Santos Ferraz RH, Rodrigues RF, Santos TC (2004). Vascular organization of the hystricomorph placenta: a comparative study in the agouti, capybara, guinea pig, paca and rock cavy. Placenta.

[B12] Wolfer J, Kaufmann P (1980). Die Ultrastruktur der Meerschweinchen-Subplazenta. Z Vet Med C Anat Histol Embryol.

[B13] King BF, Tibbitts FD (1976). The fine structure of the chinchilla placenta. Am J Anat.

[B14] Oduor-Okelo D (1984). An electron microscopic study of the chorioallantoic placenta and the subplacenta of the cane rat (*Thryonomys swinderianus *Temminck). Placenta.

[B15] Bonatelli M, Carter AM, Machado MR, De Oliveira MF, de Lima MC, Miglino MA (2005). Placentation in the paca (*Agouti paca *L). Reprod Biol Endocrinol.

[B16] Oliviera MF, Carter AM, Bonatelli M, Ambrosio CE, Miglino MA (2006). Placentation in the rock cavy, *Kerodon rupestris *(Wied). Placenta.

[B17] Nanaev A, Chwalisz K, Frank HG, Kohnen G, Hegele-Hartung C, Kaufmann P (1995). Physiological dilation of uteroplacental arteries in the guinea pig depends on nitric oxide synthase activity of extravillous trophoblast. Cell Tissue Res.

[B18] Clausen HV, Larsen LG, Carter AM (2003). Vascular reactivity of the preplacental vasculature in guinea pigs. Placenta.

[B19] Kaufmann P, Benirschke K Guinea pig *Cavia porcellus*. Comparative Placentation.

[B20] Zaki N, Kadyrov M, Huppertz B, Korr H, Kaufmann P, Mess A (2005). Trophoblast invasion can be studied *in vivo *in the subplacenta of caviomorph rodents. Placenta.

[B21] Bosco C, Buffet C, Bello MA, Rodrigo R, Gutierrez M, Garcia G Placentation in the degu (Octodon degus): Analogies with extra subplacental trophoblast and human extravillous trophoblast. Comp Biochem Physiol A Mol Integr Physiol.

[B22] Roberts CM, Perry JS (1974). Hystricomorph embryology. Symp Zool Soc Lond.

[B23] Thorburn GD, Challis JRG, Robinson JS, Wynn RM (1977). Endocrine control of parturition. Biology of the Uterus.

[B24] Heap RB, Ackland N, Weir BJ (1981). Progesterone-binding proteins in plasma of guinea-pigs and other hystricomorph rodents. J Reprod Fertil.

[B25] Tam WH (1977). Steroid synthesis in vitro by the placenta of the guinea-pig, and progesterone concentrations in systemic and uterine plasma. J Endocrinol.

[B26] Burgess SM, Tam WH (1978). Ultrastructural changes in the guinea-pig placenta, with special reference to organelles associated with steroidogenesis. J Anat.

[B27] Heap RB, Illingworth DV (1974). The maintenance of gestation in the guinea pig and other hystricomorph rodents: changes in the dynamics of progesterone metabolism and the occurrence of progesterone-binding globulin (PBG). Symp Zool Soc Lond.

[B28] Perrot-Applanat M, David-Ferreira JF (1982). Immunocytochemical localization of progesterone-binding protein (PBP) in guinea-pig placental tissue. Cell Tissue Res.

[B29] Maston GA, Ruvolo M (2002). Chorionic gonadotropin has a recent origin within primates and an evolutionary history of selection. Mol Biol Evol.

[B30] Nakav S, Jablonka-Shariff A, Kaner S, Chadna-Mohanty P, Grotjan HE, Ben-Menahem D (2005). The LHβ gene of several mammals embeds a carboxyl-terminal peptide-like sequence revealing a critical role for mucin oligosaccharides in the evolution of lutropin to chorionic gonadotropin in the animal phyla. J Biol Chem.

[B31] Bambra CS, Lynch SS, Foxcroft GR, Robinson G, Amoroso EC (1984). Purification and characterization of guinea-pig chorionic gonadotrophin. J Reprod Fertil.

[B32] Sherman GB, Heilman DF, Hoss AJ, Bunick D, Lund LA (2001). Messenger RNAs encoding the β subunits of guinea pig (Cavia porcellus) luteinizing hormone (gpLH) and putative chorionic gonadotropin (gpCG) are transcribed from a single-copy gpLH/CGβ gene. J Mol Endocrinol.

[B33] Thorburn GD (1992). The placenta, PGE_2 _and parturition. Early Hum Dev.

[B34] Ball KT, Gunn TR, Power GG, Asakura H, Gluckman PD (1995). A potential role for adenosine in the inhibition of nonshivering thermogenesis in the fetal sheep. Pediatr Res.

[B35] Crossley KJ, Nicol MB, Hirst JJ, Walker DW, Thorburn GD (1997). Suppression of arousal by progesterone in fetal sheep. Reprod Fertil Dev.

[B36] Mossman HW (1937). Comparative morphogenesis of the fetal membranes and accessory uterine structures. Contrib Embryol Carnegie Inst.

[B37] Tibbitts FD, Birge WJ (1961). Observations on the giant cells of the chinchilla placenta. Trans Illinois Acad Sci.

